# Between myopathy and mortality: Challenges in the diagnosis of *PLOD1*-related kyphoscoliotic Ehlers-Danlos syndrome

**DOI:** 10.1177/22143602261457531

**Published:** 2026-08-29

**Authors:** Katharina Vill, Theresa Brunet, Melanie Brugger, Matias Wagner, Matthias Baumann, Ulrich Schatz, Astrid Pechmann, Iris Hannibal, Moritz Tacke, Cecilia Giunta, Walter Wohlgemuth, Moritz Wildgruber, Wolfgang Müller-Felber, Astrid Blaschek, Marianne Rohrbach, Veronika Huf

**Affiliations:** 1 Department of Pediatrics I, Division of Pediatric Neurology I MUC iSPZ Hauner - Munich University Center for Children with Medical and Developmental Complexity, Dr. von Hauner Children’s Hospital, LMU University Hospital, Munich, Germany; 2 School of Medicine and Health, 9184 Institute of Human Genetics, Technical University of Munich, Munich, Germany; 3 German Research Center for Health and the Environment (GmbH), Helmholtz Zentrum München, Institute of Neurogenomics, Munich, Germany; 4 Department of Pediatrics I, Division of Pediatric Neurology, 155818 Medical University of Innsbruck, Innsbruck, Austria; 5 Department of Neuropediatrics and Muscle Disorders, Faculty of Medicine, 14879 Medical Center - University of Freiburg, Freiburg, Germany; 6 Connective Tissue Unit, Division of Metabolism and Children’s Research Center, 27243 University Children’s Hospital Zurich and University of Zurich, Zurich, Switzerland; 7 Clinic and Policlinic of Radiology, 9176 Martin-Luther University Halle-Wittenberg, Halle (Saale), Germany; 8 Department for Radiology, LMU University Hospital, Munich, Germany; 9 Department of Pediatric Radiology, Catholic Children’s Hospital Hamburg Wilhelmstift, Hamburg, Germany

**Keywords:** kyphoscoliotic Ehlers-Danlos syndrome, PLOD1-related kEDS, congenital myopathy, PLOD1, Ehlers-Danlos

## Abstract

**Objective:**

PLOD1-related kyphoscoliotic Ehlers-Danlos syndrome (kEDS) is a rare autosomal recessive connective tissue disorder characterized by generalized joint laxity, severe congenital hypotonia, progressive kyphoscoliosis, hyperextensible and easily bruised skin, ocular abnormalities, and significant vascular complications.

**Methods:**

We report on nine patients from seven families, eight of them carrying the common homozygous duplication of exons 10-16 in *PLOD1*. Longitudinal clinical assessments included muscle ultrasound (n=6) and vascular ultrasound (n=4). Genetic diagnostics varied, with most patients ultimately undergoing trio exome or genome sequencing. Urine pyridinoline analysis was performed in seven of nine patients. A literature review and age-stratified recalculation of vascular incidence, including our cohort, were conducted.

**Results:**

Diagnosis was challenging in five families, as the exon 10–16 duplication often escaped detection due to its high allele frequency. Seven patients were initially diagnosed with congenital myopathy. Muscle ultrasound revealed abnormalities in five of six sonographically examined cases. Severe vascular events included neonatal intracranial hemorrhage, fatal aortic aneurysm rupture at the age of 13 years, multiple aneurysms/dissections (ages 14–19 years), and mesenteric dissection at the age of 10 years. Four younger patients (aged 3–9 years have had no vascular complications to date. Urine pyridinoline analysis was abnormal in all tested cases.

**Discussion:**

*PLOD1*-related kEDS often presents with a phenotype of congenital myopathy, complicating genetic diagnosis and potentially leading to underdiagnosis - especially in cases where the common *PLOD1* duplication may be missed by strict frequency filters in exome or genome sequencing data. Literature and our data indicate a vascular event incidence from childhood age of ∼25%.

## Introduction

*PLOD1*-related kyphoscoliotic Ehlers-Danlos syndrome (*PLOD1*-kEDS,^1,2^ OMIM **#**225400) is a rare connective tissue disorder characterized by hypotonia, generalized joint hypermobility, early-onset kyphoscoliosis, skin fragility, ocular abnormalities, and potential cardiovascular complications. Cognitive function is typically normal. The disease is inherited as an autosomal recessive trait and is thought to be completely penetrant. The incidence has been estimated in the literature to be approximately 1:100.000 live births,^3^ but due to the rarity of the condition, its true prevalence remains largely unknown.^3,4^ While life expectancy may be within the normal range, individuals with this condition are at increased risk of life-threatening arterial ruptures and spontaneous dissections,^5^ and surgical or interventional treatment of vascular complications also carries an increased risk.^3,6^ Vascular events have been reported from early childhood to adulthood; however, due to the rarity of the disorder, the exact incidence of vascular events remains unclear.^3^ *PLOD1* encodes lysyl-hydroxylase 1 (LH1), an enzyme that hydroxylates lysyl residues within the Xaa-Lys-Gly tripeptide motifs of collagen. These hydroxylated residues are then glycosylated through the addition of galactose or glucosylgalactose units. These modifications are crucial for collagen fibril integrity and overall tissue stability. Therefore, LH1 deficiency results in insufficient hydroxylation and glycosylation of lysyl residues in the collagen helical domain, impairing cross-linking and leading to mechanical instability of connective tissue. Patients with LH1 deficiency exhibit a significantly increased urinary ratio of lysylpyridinoline to hydroxylysylpyridinoline (LP/HP) due to defective hydroxylation of collagen lysyl residues. An increased ratio of urinary total LP to HP is diagnostic for *PLOD1*-kEDS.^7–9^ More than 20 distinct pathogenic variants in *PLOD1* (originally described as *LH1*) have been linked to LH1 deficiency and the clinical presentation of *PLOD1*-kEDS. Two pathogenic variants in *PLOD1* (NM_000302.4) stand out: a 8.9 kb duplication of exons 10–16 caused by homologous recombination of intronic Alu sequences and a nonsense variant, p.Tyr511Ter in exon 14. These variants likely originated from a common ancestral gene^10^ and are two of the most common pathogenic variants in the context of *PLOD1*-kEDS.

Current surveillance recommendations for individuals with kEDS include annual physical therapy assessments for weakness and motor issues, as well as orthopedic evaluations for the management of kyphoscoliosis and recurrent dislocations. Osteopenia assessments are advised as needed, beginning between the age of 10 and 12 years. Respiratory complications should be evaluated when clinically indicated. Cardiovascular monitoring includes echocardiography at the time of diagnosis or by age 5 years, followed by full visualization of the aorta (via CT or MRA) by young adulthood, or earlier if dilatation is detected. Additionally, annual ophthalmologic examinations and screenings for inguinal hernia are recommended.^5^

We present nine pediatric cases of *PLOD1*-related kEDS from seven families, most initially presented with a clinical phenotype suggestive of a primary congenital myopathy before the broader systemic features became apparent. Eight patients carried the same homozygous duplication of exons 10–16 which was difficult to detect by whole exome/whole genome sequencing in 5 out of 6 families, suggesting that *PLOD1*-kEDS may be underdiagnosed. Since four of our patients had a history of neonatal hemorrhage, arterial rupture or dissection in infancy, indicating that this patient population is at particularly high risk for vascular events, even in childhood and young adulthood, we assessed additionally the incidence of early vascular complications among published individuals with *PLOD1*-related kEDS.

## Methods

### Inclusion criteria

Inclusion criteria were genetic evidence of *PLOD1*-related kEDS at one of the four centers in Munich and Freiburg, Germany, Innsbruck, Austria, and Zurich, Switzerland. A retrospective analysis was performed on the data collected in the clinical context.

### Clinical assessment

Patients were regularly evaluated during routine visits to the pediatric neurology and metabolic centers, with comprehensive clinical, orthopaedic, and internal medicine examinations.

### Sonographic assessment of the musculature

Longitudinal muscle ultrasound was performed in 6/9 cases and was analyzed as previously described by the authors.^8^ Muscle ultrasound was performed on the rectus femoris, vastus intermedius, tibialis anterior, gastrocnemius, deltoid, biceps brachii, triceps brachii, adductor longus, gracilis, medial hamstrings, biceps femoris, rectus abdominis, and lumbar erector spinae muscles.

Muscle imaging was graded visually. An experienced investigator classified the muscle as normal, with dark background echogenicity and clearly visible delicate internal septa, or abnormal, showing increased background echogenicity due to intramuscular fat and connective tissue, which appear bright on ultrasound.

### Genetic testing

For patient 1.I, patient 1.II proband-only exome sequencing (analysis for 1.I and 1.II together under the assumption of the same disease) and for individuals 2.I, 3.I and 5.I, trio exome sequencing of probands and their healthy parents was performed as follows: exonic DNA fragments were enriched using the SureSelect Human All Exon Kit 60Mb V6 (Agilent, Santa Clara, USA). The prepared libraries were subsequently sequenced as 100 base pair paired-end reads on a NovaSeq machine (Illumina, San Diego, USA) to an average of more than 107-fold coverage with >97% of target sequences being covered >20-fold. Alignment of reads is to the Human Genome Assembly GRCh37 (hg19). Variant calling was done using the software tools Genome Analysis Toolkit 4 (GATK 4.2.3.0), Pindel and ExomeDepth for the detection of copy number variations (CNVs). The filtering and prioritization of variants was done using the in-house software EVAdb (github.com/mri-ihg/EVAdb).

For individual 4.I trio genome sequencing was performed. DNA was prepared for sequencing using the Illumina DNA PCR-Free Library preparation kit and subsequently sequenced on the NovaSeq6000 (Illumina, San Diego, USA) to an average of greater than 52-fold coverage as 150 base pair paired-end reads. >98% of target sequences including mitochondrial DNA are covered at least 20-fold. Alignment of reads is to the Human Genome Assembly GRCh37 (hg19). Variant calling for single nucleotide variants and small Indels up to 20 bp was used as described above. Structural variants were called by a combination of different software tools and compiled by an in-house algorithm (github.com/mri-ihg/ngs_pipeline).

Individual 5.II received targeted MLPA analysis of *PLOD1* (P359-A3, MRC Holland, Amsterdam, Netherlands).

In individual 6.I mutation analysis following urinary pyridinoline measurement was performed by PCR amplification of the *PLOD1* genomic region between intron 9 and intron 16 and the result was confirmed on a second independently amplified PCR amplicon according to the protocol published in: Giunta et al., Mol Genet Metab 86:269-276, 2005.^11^

Individual 7.I received Array CGH which stayed inconclusive at the age of one year. Genome sequencing at the age of 15 years revealed a large (4.8 kb) homozygous deletion on chromosome 1, including exon 1 of *PLOD1.*

### Vascular assessment

#### Sonographic assessment of the vessels

Vascular ultrasound was performed in 4/9 cases using a sector (6 MHz) and high-frequency linear (16 MHz) transducer (GE LOGIQ E Machine, GE Healthcare. Chalfront St Giles, Buckinghamshire, Great Britain) to systematically evaluate the major arterial and venous structures.

The abdominal ultrasound included assessment of the major retroperitoneal vessels with the aorta, celiac trunk, superior mesenteric artery, renal artery origins, and iliac arteries bilaterally. Parenchymal organs were also assessed with Doppler imaging of the hilar vessels. In addition, the course of the mesenteric vessels as well as abdominal wall vasculature were assessed using the high-frequency 16 MHz transducer.

A comprehensive ultrasound examination of the major vessels of both lower extremities was performed from the inguinal region to the ankle joint, including both superficial and deep vessels. A transverse scanning approach was used with probe compression to assess venous compressibility whilst ensuring clear visualisation of the arteries without interference or dilatation. The muscle compartments were then systematically scanned transversely to identify any vascular irregularities or deviations in vessel spacing.

Bilateral carotid and vertebral arteries were assessed, as well as a view into the upper mediastinum. A systematic evaluation of the bilateral subclavian arteries, brachial arteries and forearm arteries up to the wrist was performed. Muscle compartments were also scanned using a transverse approach to identify any vascular irregularities. Notably, Doppler imaging of the extremities was not routinely included in this protocol.

### MR examination

In case of sonographic abnormalities MR Angiography of the affected vessel was executed. Furthermore, MRI of the brain with contrast-medium was performed in patients 1.I, 2.I, and 3.I.

#### Cardiology

All surviving patients had a cardiovascular evaluation every year including echocardiography.

#### Urinary pyridinoline analysis

The biochemical urinary pyridinoline cross-link markers lysyl-pyridinoline (LP or deoxypyridinoline DPD) and hydroxylysyl-pyridinoline (HP or pyridinoline PYD) are well-established indicators of osteoclastic bone resorption and collagen breakdown. An increased ratio of LP to HP is diagnostic for *PLOD1*-kEDS.^8^ Total urinary pyridinolines were measured as described.^9,12^

## Results

**Figure 1.**
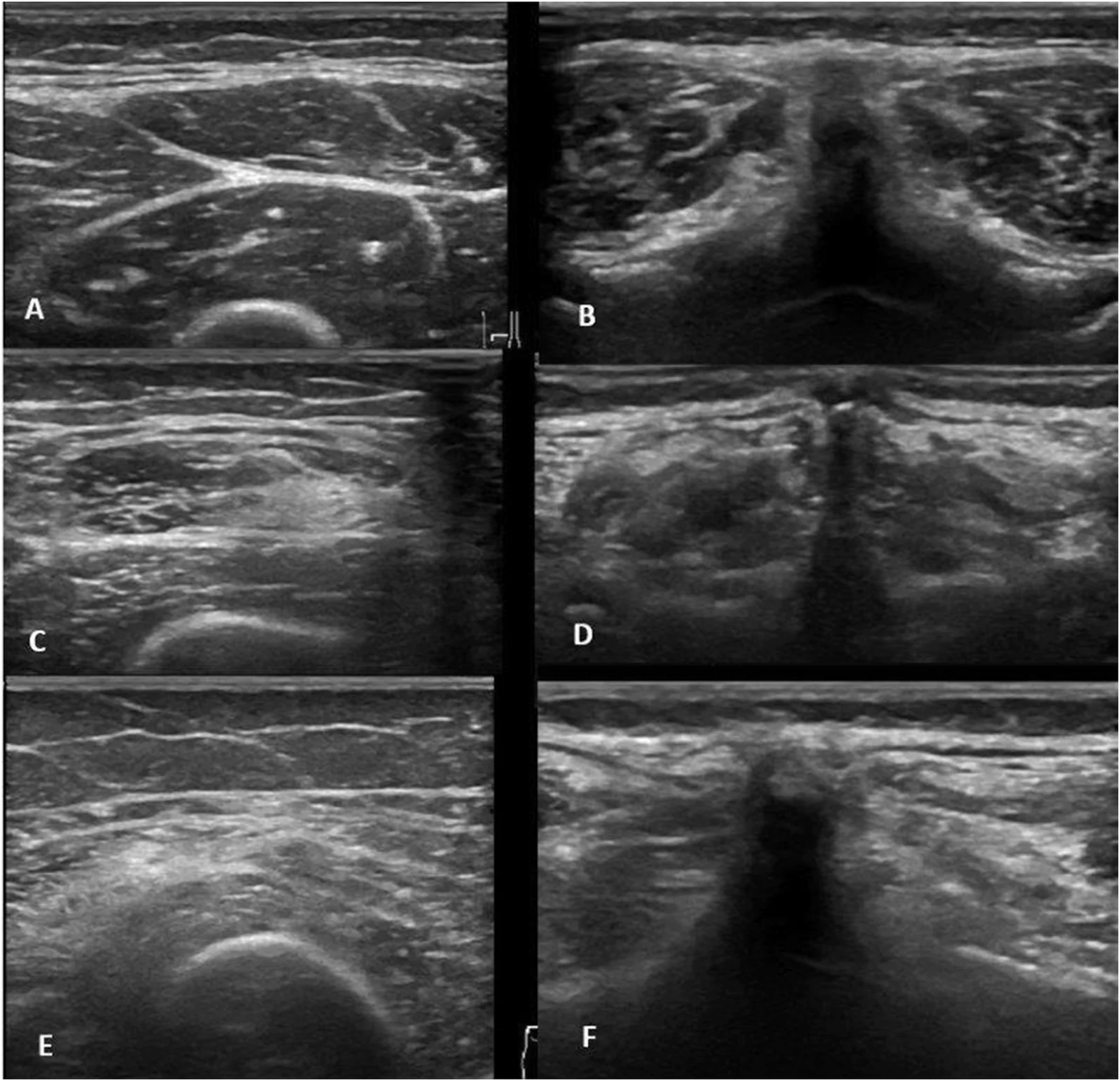
Quadriceps femoris muscle (A, C, E) and lumbar erector spinae muscle (B, D, F) of a healthy control (age 4 years, A, B), patient 3.I (age 5 years, C, D), patient 1.I (age 13 years, E) and patient 3.I (age 9 years, F): Marked, partly string-like or knotty, partly diffuse increase in echogenicity of the musculature in kEDS patients compared to healthy controls.

Detailed case reports and family trees are provided in the Supplementary Material, and a tabulated summary is given in Table 1. Nine patients (three males and six females) from seven families (two pairs of cousins) presented with various symptoms, including pronounced muscular hypotonia and joint hypermobility, and skeletal abnormalities such as kyphosis and/or scoliosis. Initial diagnoses suggested congenital myopathy in six out of seven families due to early muscle weakness or hypotonia (not only functional, but also formal muscle weakness in the first years of life in most cases) and delayed motor development, as well as abnormal muscle ultrasound in five out of six patients on which ultrasound was executed with increased echogenicity of different muscle groups (see Figure 1). In one patient aged 1.5 years muscle biopsy revealed a pronounced myopathic pattern with marked fiber caliber variability and a slight increase in interstitial connective tissue; morphologically, these findings were compatible with congenital myopathy. However, as the patients grew older, joint hypermobility and connective tissue fragility became more prominent features, and formal strength testing became increasingly normal.

**Table 1.** Clinical Features of the patients included in this study. Yes/apparent (+), no/absent (-) n.a. (not performed). The vascular findings in brackets are unspecific and could in general be associated to a vascular event, but were not definitively linked to one in these patients —and certainly not to a severe event.

**Patient (sex)**	**Age at last examination**	**Age at independent walking**	**Motor delay**	**Muscular hypotonia and/or weakness**	**Joint hyper-mobility**	**Kypho-scoliosis**	**Hypertrophic scarring**	**Vascular complications**	**Orthopedic**
I.1 (m)	19 years	n.a.	+	+	+	Kyphosis 60 degrees	+	Dissection of the superior mesenteric artery; harmonic dilation of the left common iliac artery; venous aneurysm and AV-fistulas the left thigh, varices with collateral circulation in the abdominal wall, aneurysm and rupture of the popliteal artery	Flat Feet, Arthrosis in ankles, pectus excavatum, protuberant lower abdomen, Osteopenia
I.2 (m)	7 years (died age 13 years)	22 months	+	+	+	-	+	+Ruptured aortic aneurysm	Internal rotated hips, flat feet, protuberant lower abdomen
2.I (f)	9 years	2,0 years	+	+	+	-	+	(Questionable aortic elongation in sonography, not confirmed by MRI)	Flat feet, slightly protuberant lower abdomen
3.I (f)	6 years	2,1 years	+	+	+	+	+	-	Flat feet, protuberant lower abdomen
4.I (f)	4 years	2,3 years	+	+	+	+	+	-	Flat feet, protuberant lower abdomen
5.I (f)	6 years	2,7 years	+	+	+	+	no	(History of thalamostriatal vasculopathy)	Flat feet
5.II (f)	4 years	2.5 years	+	+	+	+	no	(Suspicion of intracranial haemorrhage at birth)	Flat feet
6.I (f)	10 years	2.2 years	+	+	+	No kyphosis, but thoracolumbar scoliosis (16°)	no	+Superior Mesenteric Artery Dissection	Scapula alata Pectus excavatum Recurrent luxation of shoulders Shortened Dig 5 hand and Dig 4 feet
7.I (m)	17 years	4 years	+	+	+	+	no	+Postpartum intraventricular hemorrhage (grade II)	Pes planovalgus

**Patient (sex)**	**Beighton score at last examination**	**Increase in echogenicity in muscle ultrasound**	**Urinary pyridinoline analysis (abnormal LP/HP ratio)**	**Congenital myopathy (differential diagnosis)**	**Difficulties in genetic diagnosis (was exome negative?)**	**Miscellanous**	**Ocular complications**		
I.1 (m)	6	+(Gracilis, rectus abdominis, quadriceps femoris, paravertebral muscles)	+	+	+NGS panel Trio-WES inconclusive	​	Strabismus concomitans convergens		
I.2 (m)	7	no	n.a.	+	+Trio-WES inconclusive	​	-		
2.I (f)	7	+(Lumbar paravertebral musculature)	+	+	Trio-WES inconclusive	​	-		
3.I (f)	7	+(Quadriceps femoris, soleus, adductor longus, gracilis, biceps brachii, paravertebral muscles)	+	+	+NGS panel Trio-WES inconclusive	​	-		
4.I (f)	7	+(Medial hamstrings, biceps femoris, gastrocnemius, gracilis, paravertebral muscles, quadriceps femoris)	+	+	+Trio-WES inconclusive	​	-		
5.I (f)	8	+(Lower legs muscles questionable increased fatty infiltration.)	+	+	Trio-WES inconclusive	Epilepsy, submucosal velum cleft, horseshoe kidney	-		
5.II (f)	9	n.a.	n.a.	-(Diagnosis indicative due to positive family history)	-(Diagnosis indicative due to positive family history)	​	Bilateral periorbital hematomas at birth		
6.I (f)	5	n.a	+	+	No problems observed	Coeliac disease, asthma	-		
7.I (m)	n.a.	n.a.	+	+	-		Blepharoconjunctivitis sicca

Vascular complications were significant in four cases, with arterial rupture and/or dissection occurring in one patient perinatally, and in three patients during their childhood or adolescence. One patient died from aortic rupture. Skeletal findings showed a highly suggestive Beighton score of 5–9 at the latest examination for all patients examined (not performed in one). Kyphosis or kyphoscoliosis was present in five patients, and all had flat feet. Hypertrophic scarring was a common finding and ocular anomalies were reported in two patients.

Eight of nine patients eventually were identified to carry a homozygous duplication of exons 10–16 in the *PLOD1* gene. Patient 7.I was identified as carrying a large (4.8 kb) homozygous deletion on chromosome 1, including exon 1 of PLOD1. Genetic diagnosis proved challenging, with five patients undergoing extensive genetic testing, including inconclusive exome and genome sequencing, before the homozygous duplication of exons 10–16 in the *PLOD1* gene was identified. The common pathogenic *PLOD1* intragenic duplication has an allele frequency (AF) in the new gnomAD structural variant data set of 0.00008 (5/59022 alleles) in the European gnomAD population (listed as DUP_CHR1_A8EB9477) and was surprisingly three times more frequent in the in-house exome database in Munich with an AF of 0.00025 (16/63350 alleles) after exclusion of kEDS cases and their parents. The AF of 0.00016 of the second most frequent causative variant in *PLOD1* (p.Tyr511Ter) indicates a reduced sensitivity for variant calling in the gnomAD dataset and possibly also in the in-house data.

Urine pyridinoline analysis supported the diagnosis by showing an abnormal elevation in the collagen LP/HP ratio in all seven patients, whose urine was tested (7/7). Detailed case reports for all patients can be found in the supplementary material.

### Literature review

The total of 110 reported cases of kEDS is derived from 84 patients summarized in the classification publication by Brady et al. (*American Journal of Medical Genetics Part C: Seminars in Medical Genetics*, 175C:70–115, 2017), supplemented by additional cases published subsequently, including the cohort presented in this study. Of these 110 patients, 33 were reported to have experienced vascular events. Details of the literature review are listed in Tables 2 and 3, with references to the respective publications.

**Table 2.** Age distribution of reported vascular event in *PLOD1*-kEDS. The manuscript reports on 33 cases with vascular complications, the age at event was available in 31/33 patients.

Age at event (years)	Number of patients with events/overall Prevalance *PLOD1*-kEDS (%)
peri/neonatal	12/110 (10.9)
0.5-6	0 (0)
7-12	4/110(3.6)
12-20	8/110 (7.3)
>20	7/110 (6.4)
All ages	33/110 (30)

**Table 3.** Literature review for vascular events in *PLOD1*-kESD. Including this manuscript, 33 cases with vascular complications have been reported; age at event was available in 31/33.

Published (year)	Publication (Reference Number)	Number of patients with vascular complications/total number of patients	age at first vascular complication (age in years)
1989	Wenstrup et al., 1989^13^	3/10	Neonatal/?/?
1998	Heim et al., 1998^14^	1/1	10
2000	Yeowell et al., 2000^10^	1/2	14
2003	Akpinar et al., 2003^15^	2/2	13/13
2005	Giunta et al., 2005^11^	2/9	neonatal/antenatal
2007	Debnath et al., 2007^16^	1/1	20
2011	Rohrbach et al., 2011^4^	2/15	neonatal and 27
2012	Gok et al, 2012^17^	1/1	12
2014	Tosun et al, 2014^18^	1/1	antenatal
2014	Busch et al., 2014^19^	1/1	32
2016	Yung et al., 2016^20^	1/1	24
2017	Quade et al., 2017^21^	1/1	13
2017	Zahed et al. , 2017^22^	2/2	neonatal
2017	Working et al., 2017^6^	1/1	8
2018	Henneton et al., 2018^3^	1/1	41
2020	Lim et al., 2020^23^	1/2	antenatal
2020	Shin et al., 2020^24^	1/1	antenatal
2021	Zhao et al., 2021^25^	1/1	neonatal
2021	Zieminski et al., 2021^26^	1/1	42
2022	Yan et al., 2022^27^	1/1	neonatal
2023	Foy et al., 2023^28^	1/1	29
2024	Bhandari et al., 2024^29^	2/3	13 and 10
2025	This manuscript, 2025	4/9	Neonatal, 10, 13 and 14

Literature review for vascular events in *PLOD1*-kEDS. Including this manuscript, 33 cases with vascular complications have been reported; age at event was available in 31/33.

Of all the reported vascular events, antenatal/neonatal brain hemorrhage was the most commonly observed complication, followed by aortic dilatation/dissection and rupture of medium-sized arteries in teenagers and adults. Younger children (aged 7–12) were less affected. Vascular insults have not yet been reported in children under 5 years of age outside of the neonatal period. A search of *ClinicalTrials.gov* revealed no ongoing or completed clinical studies investigating specifically *PLOD1*-related kEDS.

## Discussion

### Diagnostic overlap between congenital myopathies and *PLOD1*-related kEDS

*PLOD1*-related kEDS is deceptively complex. In the literature, congenital myopathy is rarely reported as a suspected diagnosis in patients affected with *PLOD1*-kEDS,^30^ in contrast to severe muscular hypotonia, which is consistently described.^30,31^ However, our data and the literature demonstrate that *PLOD1*-kEDS can closely resemble congenital myopathy in early childhood due to overlapping clinical features, including not only functional but also formal muscular weakness and delayed motor milestones. This frequently results in referral to a neuromuscular center, as was the case for the majority of families in our study. Furthermore, muscle ultrasound may reveal an abnormal myopathic pattern, as observed in five out of six sonographically examined cases in our cohort (Figure 1).

The findings of the muscle biopsy patient 7.I were compatible with congenital myopathy. In conjunction with the myopathic ultrasound findings in the majority of cases, this indicates that the muscular involvement in *PLOD1*-kEDS extends beyond a mere phenotypic mimicry of congenital myopathy and represents a genuine myopathic disease pattern in a relevant number of patients. However, this myopathic nature of the disorder is not yet widely recognized within the neuromuscular field, and *PLOD1*-related kEDS is therefore often not perceived as falling within the core spectrum of neuromuscular diseases.

Because creatine kinase (CK) levels are typically normal in *PLOD1*-kEDS, as was the case in all of our patients, individuals may initially be suspected of having *COL6*-related disorders, such as Bethlem or Ullrich congenital muscular dystrophy, or congenital myopathies with structural abnormalities rather than muscular dystrophies. In addition, mild connective tissue involvement in some congenital myopathies, particularly with distal manifestations as seen in Bethlem and Ullrich disease, may further contribute to diagnostic confusion. *FKBP14*-related kEDS represents another important differential diagnosis with highly overlapping clinical features, including muscular weakness reported in up to 63% of affected individuals.^32^

When conducting a diagnostic evaluation in a neuromuscular center, it is crucial to note that *PLOD1* is not included in most commercial neuromuscular gene panels, further underscoring the diagnostic challenges faced by clinicians.

### Vascular risks and the importance of early assessment

Orthopedic and ophthalmologic complications in *PLOD1-*kEDS are evident, even if genetic diagnosis is not pursued,^14^ and will generally be addressed accordingly. However, the high vascular risk remains a major challenge, even when the diagnosis is established.^3,9,11,19,28^ Without genetic confirmation, vascular insults may occur unexpectedly due to a lack of awareness of these life-threatening complications. The exact number of affected children is not known. A 2018 case report describes a 41-year-old woman with recurrent arterial dissections and noted that her younger sister died at the age of 9 years from a spontaneous abdominal aortic rupture,^3^ highlighting the variability in the onset of vascular complications.^4^ Recent reports increasingly document vascular insults in children.^3,5,29^ In our cohort of nine patients with *PLOD1*-related kEDS, vascular complications were observed in four patients. Patient 1.I experienced multiple vascular events, including an arterial popliteal rupture at the age of 14 years and mesenteric dissection aged 16 years, successfully treated by interventional stent implantation in his teens, while patient 1.II died of complications from a ruptured aortic aneurysm at the age of thirteen years and patient 6.I experienced a mesenteric dissection (conservatively managed) at the age of ten years. Patient 7.I had postpartum intraventricular hemorrhage (grade II) on the right side. This incidence is consistent with the newly calculated prevalence of vascular complications in published cases of *PLOD1*-related kEDS, where 33 of 110 patients (30%) experienced such complications. It is striking that the age of onset is highest in the neonatal/perinatal period, probably due to the mechanical effects of birth, but also >25% in childhood from the age of seven years, making this a childhood disease that needs to be addressed early by the treating physician, most commonly the neuropediatrician.

Vascular ruptures and dissections may arise spontaneously, typically without a clearly identifiable triggering factor. Arterial rupture is a risk for early death in kEDS but early vascular assessment could help reduce the severity of complications. Our data, supported by case report of patient 1.I, suggest that vascular insults in *PLOD1*-related kEDS might be preceded by aneurysm formation rather than spontaneous vessel rupture, as seen in vascular EDS (*COL3A1*-related vEDS).^33^ This indicates that kEDS may involve progressive weakening of the vascular wall, leading to aneurysm development before rupture occurs. If this is the case, early aneurysm detection can allow for preventive intervention, potentially reducing life-threatening events. However, catheter examinations and interventions should only be performed if absolutely necessary by dedicated specialists for this patient group, as the risk for complications is extemely high due to their fragile vessels.^6,19^ ^34^ However, in cases of critical aneurysms or dissections, there may be no alternative. It is also unclear at what age the risk of interventions rises disproportionately due to the degenerative process of the vessels.

### Genetic diagnostic challenges

Detecting the common 8.9kb duplications of exons 10-16 in short read NGS data can be challenging due to several reasons. Exome sequencing is optimized for detecting single nucleotide variants (SNVs) and small insertions or deletions (indels). However, these methods often struggle to accurately detect larger structural variants, including exon duplications, particularly if the duplications are intragenic (within a single gene) (PMID: 35191116). In clinical settings, sequencing data is filtered to exclude common variants based on population databases or in-house controls, assuming that common variants are unlikely to be pathogenic. Strict filtering criteria might mask these variants with a high allele frequency, such as the common 8.9kb duplication (PMID: 28306225). Furthermore, exon duplications are typically harder to detect than whole-gene duplications because they involve smaller, specific segments of the gene. Detecting exon duplications requires specialized CNV (copy number variation) detection tools that can identify changes in copy number. Many standard CNV tools have limited sensitivity when the duplicated region is small or if the duplication spans only a few exons, especially if coverage is low or uneven (PMID: 39669630). NGS coverage may not be uniform across all exons, and duplications may be missed if there is insufficient depth of coverage in the duplicated region. Additionally, reduced specificity leads to false positive calls which in turn lead to a falsely increased in-house frequency. Both effects can lead to the duplication being overlooked or excluded by strict filtering criteria during variant prioritization. To overcome these challenges, more specialized bioinformatics tools designed for detecting structural variants or exon-level duplications may be used. Importantly, the clinical phenotype of the patient is crucial in guiding the genetic analysis and interpreting the results, as it can help prioritize the search for specific variants associated with the observed clinical features. Clinicians and geneticists must be more aware of EDS as a differential diagnosis for myopathies and of the *PLOD1* duplication as a frequently overlooked variant.

Arterial and venous complications, including life-threatening arterial dissections and aneurysms, are not only a hallmark of *PLOD1*-related kEDS, but also of *FKBP14*-related kEDS.^23,35^ In the previous manuscripts, approximately 30% of the patients described had vascular abnormalities. In the family with the initial index patient of three patients (of which one patient only had clinical *FKBP14*-related kEDS, not genetically confirmed), one patient died of a ruptured aortic aneurysm at the age of twelve.^32,35^ This further underlies the overlapping clinical features of the two genetic forms of kEDS.

Urinary pyridinoline analysis provides a biochemical tool with published diagnostic character,^8,12^ however this test is only performed when clinical suspicion exists, further emphasizing the need for greater awareness of *PLOD1*-kEDS among clinicians. Urinary LP/HP ratio can either exclude or confirm the diagnosis of *PLOD1*-kEDS and suggest FKBP14-kEDS based on strong clinical signs suggestive for kEDS.

### Ultrasound as a first-line screening tool for vascular abnormalities?

Given the progressive nature of vascular complications in kEDS, regular vascular surveillance seems essential. However, the decision to undertake invasive or interventional therapy must be made with extreme caution. The need for regular echocardiograms is well recognized. We also recommend performing annual magnetic resonance angiography of the brain. Although only isolated vascular insults in the brain have been reported to date, these are likely to be fatal, and the current patient group is small.

In experienced hands, vascular ultrasound serves as an accessible, cost-effective, and non-invasive bedside tool that can precede MRI angiography screenings. Particularly in children, who typically provide an excellent acoustic window, ultrasound may in some cases partially replace MRI, reducing the need for frequent sedation or anesthesia. However, ultrasound reliability depends on the examiner’s experience, emphasizing the importance of assessments being performed at centers with expertise in connective tissue disorders.

While ultrasound is a valuable screening tool, it cannot entirely replace MRI or CT angiography, particularly for assessing deeper vascular structures such as the aorta or visceral arteries. However, it remains an excellent first-line modality for routine monitoring in children, enabling early detection of vascular changes and guiding advanced imaging decisions.

### Therapeutic management: The need for specialized centers and emergency transport

Vascular abnormalities requiring intervention should be managed exclusively at centers with experience in complex vascular complications of connective tissue disorders. The risk of complications remains elevated in this scenario, too, but in the hands of inexperienced individuals, the outcome is likely to be unfavorable.

In cases of suspected vascular rupture, dissection or aneurysm-related complications, patients should be transported directly to a specialized center familiar with their condition. This is especially critical in the emergency setting, where mismanagement or delay can be fatal. If a life-threatening vascular event is suspected, immediate and rapid transport to a designated vascular or connective tissue disease center should be prioritized to ensure optimal care.

### Incidence and the need for preventive strategies

The overall incidence of vascular events in *PLOD1*-kEDS is high, with a significant increase in risk during adolescence. A re-examination of all previously reported cases,^5^ combined with the patients in this manuscript, indicates that a substantial proportion of affected individuals in young adulthood (aged 12-42 years, 14% of all reported cases from the age of 12 years) succumb to vascular events, primarily involving large arteries such as the aorta. The popliteal arteries also appear to be at increased risk.

These findings highlight the need for early and regular vascular surveillance, particularly in adolescents. Preventive measures, including vascular imaging and proactive aneurysm management, may help reduce mortality. Our data suggest that ultrasound, when performed by an experienced examiner, could serve as an alternative to MRI or CT for imaging medium-sized arteries. This approach is less invasive and more cost-effective for both patients and healthcare systems. A structured vascular monitoring strategy is essential to detect aneurysms before rupture, allowing timely intervention if inevitable. However, it is still unclear to what extent the risk of interventions and surgical procedures increases with age. Common sense might suggest this, given the degenerative process of the vessel walls discussed above.

### Significance of beta-blocker therapy

Since there is currently no causal treatment and surgical and interventional therapies are at high risk, early initiation of beta-blocker therapy could serve as a preventive measure to reduce hemodynamic stress on the vascular system, as suggested in vascular-EDS.^5,36^ This recommendation is often made, but there are no data on therapy in the pediatric population. Given the high risk of arterial complications, beta-blockers may help to reduce vascular events by decreasing arterial wall tension, as has been suggested for other connective tissue disorders involving the vasculature. Due to the high incidence in children and adolescents, we are inclined to offer generous therapy based on blood pressure percentiles from childhood, aiming for values in the range of 3rd–25th percentile, given the limited treatment options. Further studies are needed to evaluate the efficacy of beta-blockers in *PLOD1*-related kEDS. Avoidance of contact sports and other high-impact sports seems essential.

## Conclusion

The complex multi-systemic nature of PLOD1-related kEDS—which often presents with a pronounced myopathic component—combined with its significant vascular risks, underscores the need for early clinical suspicion to prevent misdiagnosis and enable timely, potentially life-saving interventions. Clinicians should remain vigilant when treating infants with severe congenital hypotonia and significant motor delays, particularly if they exhibit pronounced joint hypermobility. Key clinical features that should prompt further testing include muscle weakness combined with normal CK levels and abnormal muscle ultrasound results showing increased connective tissue inside some skeletal muscles, as well as early vascular warning signs such as neonatal or antenatal hemorrhage. Additional progressive kyphoscoliosis and hypertrophic scarring can be also indicative.

Heightened awareness of its clinical presentation and vigilant vascular monitoring, especially during adolescence, are essential for improving patient outcomes. The high mortality rate further emphasizes the urgent need for effective causal therapies. In light of recent advances in genetic research, prioritizing the development of gene-based therapies for this disorder is imperative.

## Supplemental material

Supplemental material - Between myopathy and mortality: Challenges in the diagnosis of *PLOD1*-related kyphoscoliotic Ehlers-Danlos syndrome Supplemental material for Between myopathy and mortality: Challenges in the diagnosis of *PLOD1*-related kyphoscoliotic Ehlers-Danlos syndrome by Katharina Vill, Theresa Brunet, Melanie Brugger, Matias Wagner, Matthias Baumann, Ulrich Schatz, Astrid Pechmann, Iris Hannibal, Moritz Tacke, Cecilia Giunta, Walter Wohlgemuth, Moritz Wildgruber, Wolfgang Müller-Felber, Astrid Blaschek, Marianne Rohrbach, Veronika Huf in Journal of Neuromuscular Diseases

## Appendix

### Abbreviations

(kEDS): kyphoscoliotic Ehlers-Danlos syndrome
(WES): whole exome sequencing
(WGS): whole genome sequencing
(LP): lysyl-pyridinoline
(HP): hydroxylysyl-pyridinoline.
