# Supplementary material for: Between myopathy and mortality: Challenges in the diagnosis of PLOD1-related kyphoscoliotic Ehlers-Danlos syndrome
Source: J Neuromuscul Dis. 2026 Aug 29:22143602261457531. Online ahead of print. doi: 10.1177/22143602261457531 (PMC13526180; doi:10.1177/22143602261457531)
Supplement: Supplementary file 1 [file sj-pdf-1-jnd-10.1177_22143602261457531.pdf]

Supplemental Case reports:

Patient 1.I (male):

Clinical Presentation and Medical History

Patient 1 was last evaluated at 18 years of age. He was born at 34 weeks' gestation as part of a twin pregnancy. Both parents are healthy, non-consanguineous, but originate from the same village. Immediately after birth, the patient exhibited pronounced muscular hypotonia, generalized joint hypermobility, and pectus excavatum. Cutis laxa was absent. At six months, muscle ultrasound revealed significantly increased echogenicity in both arms and legs. Muscle biopsy demonstrated fiber-type disproportion, leading—together with the clinical findings—to the diagnosis of congenital myopathy.

From school age onward, the clinical picture increasingly suggested a disorder within the Ehlers-Danlos spectrum, with joint hyperlaxity as the predominant feature. Intermittent occlusion therapy was performed for strabismus. Despite initial global developmental delay, cognitive development proceeded normally and there was no loss of motor milestones.

Motor and Skeletal Findings

At one year of age, thoracic kyphosis became apparent, prompting initiation of corset therapy at age two. Motor skills gradually improved: assisted ambulation was possible at 2.5 years, independent walking at 3 years, and stair climbing at 3.5 years. The patient developed hypertrophic scar formation and received long-term orthotic treatment. A protuberant lower abdomen was noted. From age 14, recurrent dislocations of the right shoulder occurred.

Orthopedic examination at age 18 revealed severe kyphosis (60°) following discontinuation of corset therapy, as well as severe, painful planovalgus deformities of the feet, persistent pectus excavatum, coxae in flexion of both hips with good femoral head coverage, bilateral genu valgum, right-sided external tibial rotation, and right-sided ankle arthrosis. Functional muscle weakness was minimal. The patient uses lower limb orthoses and is able to ambulate independently, though he experiences foot pain even with orthoses.

Genetic Diagnosis

Molecular confirmation remained elusive for many years, including after exome sequencing of both, him and his affected cousin (patient 1.II) in 2015. Ultimately, in 2019, breakpoint PCR according to Pousi et al. (1994) and MLPA analysis identified a deletion of exons 10–16 in the *PLOD1* gene, which was retrospectively confirmed in the exome data.

Muscle Ultrasound

At six months, muscle ultrasound showed markedly increased echogenicity in the arms and legs. At age 18, the gracilis muscles demonstrated mild increased background echogenicity with preserved intramuscular septa. More pronounced changes were observed in the rectus abdominis (mild to moderate alterations) and in the quadriceps

femoris and paravertebral muscles (moderate echogenicity increase and reduced septal definition). The gastrocnemius, tibialis anterior, adductor longus, medial hamstrings, biceps femoris, biceps brachii, triceps brachii, and deltoid muscles exhibited normal echotexture.

Urinary pyridinoline analysis (nmol/mmol creatinine, HPLC):

- Pyridinoline/creatinine (HP): 35
- Deoxypyridinoline/creatinine (LP): 216.71
- LP/HP ratio: 6.19
  Reference values for age 15–17 years: HP 42–200, LP ≤59; for age 11–14 years: HP 349 ± 148, LP 66 ± 28 (n=13;). The markedly elevated LP/HP ratio and abnormal collagen cross-linking values confirm the diagnosis.

Vascular Complications, and Outcomes

At 13.5 years, the patient experienced a suspected arterial rupture in the right popliteal fossa, presenting with acute pain, syncope, and massive swelling. The event was initially misdiagnosed externally; weeks later, sonography revealed a persistent hematoma. Annual external cardiac monitoring was performed from age 14 by transthoracic echocardiography. At 16 years, the patient suffered a dissection of the superior mesenteric artery, managed by interventional radiological stent placement. A venous aneurysm in the left thigh, which was progressive in size and accompanied by two arteriovenous fistulas connecting to the femoral artery, was surgically opened at age 17. However, due to extreme vessel fragility and the risk of exsanguination, the intervention was ultimately abandoned. Multiple collateral vessels developed in the left thigh, displacing genital structures and forming extensive abdominal wall collaterals; erectile function remained intact.

Vascular Ultrasound (Age 18)

- Left lower extremity: The left common femoral artery is significantly dilated below the inguinal ligament (8 mm vs. 5 mm on the right). The left femoral artery measures nearly 4 cm in the adductor canal and exhibits marked flow turbulence. The artery courses ventromedially to the venous aneurysm. The left great saphenous vein is dilated.
- Right lower extremity: The popliteal artery is difficult to visualize, likely due to a residual hematoma from two years prior. No vascular anomalies are seen in the lower leg.
- Neck: The left jugular vein is locally enlarged; all other vessels are unremarkable.
- Arms: No vascular pathology bilaterally.

Contrast-Enhanced MR Angiography of the Thoracoabdominal Aorta (Age 18)

- Left subclavian artery: A circumscribed area without contrast enhancement, most likely a pseudo-occlusion; otherwise, unremarkable morphology.
- Thoracic aorta and abdominal vessels: A stent is present in the superior mesenteric artery, but the lumen cannot be assessed due to artifacts; the vessel is tortuous. The left iliac artery is more pronounced than the right. Multiple collateral vessels are visible in the abdominal wall.

Abdominal findings: Compression of the left liver lobe by pectus excavatum; otherwise, normal liver parenchyma. A 2.5 cm renal cyst is present at the upper pole of the right kidney. The abdomen was not fully visualized.

<u>MR Angiography of the Pelvic and Lower Limb Vessels with Contrast (Age 18)</u>

In the lumbar region, there is an unclear cystic lesion lateral to the right fifth lumbar vertebra, possibly a lumbar artery aneurysm.

In the left pelvis and lower extremity, there is a harmonious enlargement of the left pelvic axis, involving both the common and external iliac arteries. The superficial femoral artery is patent bilaterally. The left common femoral vein is occluded, with venous drainage occurring via collateral pathways (internal femoral vein, deep femoral vein, and abdominal wall varices). A lobulated venous aneurysm measuring 30 × 33 × 64 mm is present in the proximal thigh adjacent to the superficial femoral artery. Two arteriovenous fistulas are suspected: one in the adductor canal between the aneurysm and the superficial femoral artery, and a second in the mid-thigh involving a branch of the superficial femoral artery and a muscular branch of the superficial femoral vein. The proximal veins demonstrate contrast enhancement in the early arterial phase. There is soft tissue hyperplasia of the left lower leg.

In the right pelvis and lower extremity, a small aneurysm (10 mm) of the tibiofibular trunk is present.

There is a bone cyst in the left ilium. More pronounced right ankle arthrosis with subchondral cysts is noted. Possible right hip dysplasia is present (assessment limited).

<u>Cranial MRI with Contrast (Age 18)</u>

There is a recurrent filling defect in the left subclavian artery, likely due to superimposition. Otherwise, the arteries of the neck and head are normal. The right transverse sinus is hypoplastic. Calvarium is markedly thickened.

Patient 1.II (male):

<u>Clinical Presentation and Medical History</u>

Patient 1.II was evaluated at ages 6 and 7 years. He was the only child of consanguineous parents (first cousins, second degree) and a first-degree cousin of patient 1.I. He was born at 36 weeks' gestation and exhibited pronounced neonatal hypotonia, necessitating nasogastric feeding during the first month of life due to poor sucking ability. Examination revealed markedly hyperextensible skin, pronounced joint hypermobility, and hypertrophic scarring.

<u>Motor and Skeletal Findings (age 7 years):</u>

Early motor milestones were significantly delayed: he achieved independent sitting at 12 months and independent ambulation at 22 months. His clinical course was initially notable for generalized muscle weakness, leading to a suspected diagnosis of congenital

myopathy, marked ligamentous laxity. At his last documented visit, the patient presented with generalized joint hypermobility, including excessive ankle dorsiflexion, internal rotation of the hips with genu varum (bow-legged stance), an unsteady gait with frequent falls, and striking hypertrophic scar formation. He had a protuberant lower abdomen. There was no significant kyphoscoliosis. Despite his motor difficulties, he was able to walk independently, ascend stairs with alternating feet, and showed no evidence of axial muscle weakness.

Genetic Diagnosis:

Initial genetic investigations, including whole exome sequencing at age seven, were inconclusive. At age eight, genetic testing identified a homozygous duplication of exons 10–16 in the *PLOD1* gene by MLPA analysis, confirming the diagnosis. This duplication was initially missed due to strict filtering criteria based on allele frequency.

Muscle Ultrasound (ages 6 and 7 years):

Muscle ultrasound demonstrated normal echogenicity in all major muscle groups (quadriceps femoris, gastrocnemius, tibialis anterior, biceps brachii, triceps brachii, and paravertebral muscles).

Vascular Imaging, Complications, and Outcomes:

No vascular ultrasound was performed during follow-up. Due to the family's residence several hundred kilometers away, the patient was lost to follow-up after his last documented visit at age seven, at which time a genetic diagnosis had not yet been established. At age 13, the patient developed severe abdominal pain followed by acute cardiovascular collapse and died from complications of a ruptured aortic aneurysm.

Urinary Pyridinoline Analysis: Not performed.

Patient 2.I (female):

Clinical Presentation and Medical History

Patient 2 was last examined at age 9 years. She was born after a gestation of 39 weeks and 4 days, with no neonatal complications. She was initially referred due to gross motor developmental delay and has been under ongoing follow-up at our center since early infancy. The predominant clinical feature has been severe connective tissue fragility without significant muscular weakness. She also had an umbilical hernia, which was surgically corrected at age two.

Motor and Skeletal Findings

Despite early motor delays, she achieved independent walking by age two. At her most recent assessment, her motor function was mildly impaired, with some incoordination during running and hopping. She could rise from the floor without a Gowers' sign, although minor truncal compensation was observed. Examination confirmed hyperextensible connective tissue, a mildly protuberant lower abdomen and flatfoot

deformity with a mildly varus calcaneus. Spinal alignment was normal, with no significant kyphoscoliosis. She exhibited mild pectus excavatum and a mildly protuberant lower abdomen.

Genetic Diagnosis

Initial exome sequencing at age two was inconclusive. Reanalysis of trio exome data, considering the recent suspicion of Ehlers-Danlos syndrome, revealed a homozygous duplication of exons 10–16 in the *PLOD1* gene. This duplication was initially missed due to strict filtering criteria based on allele frequency.

Muscle Ultrasound

Longitudinal ultrasound showed moderate increased echogenicity in the paravertebral lumbar muscles (erector spinae), while other muscle groups appeared largely normal.

Vascular Imaging, Complications, and Outcomes

A comprehensive vascular ultrasound demonstrated open, compressible major arteries and veins in the lower limbs. A questionable irregular contour was noted in the left posterior tibial artery, but no aneurysm was identified. Abdominal ultrasound was unremarkable, aside from a suspicion of minimal aortic elongation. Subsequent MRI showed no pathological findings: the ascending aorta measured 1.8 cm, and the thoracic aorta 1.0 cm in diameter, within normal limits.

Urine pyridinoline analysis (in nmol/mmol creatinine) (HPLC measurement):

(HP) pyridinoline/creatinine: 68; (LP) deoxypyridinoline/creatinine: 332.6; LP/HP ratio: 4.89. The age-related reference values for collagen cross-linking were abnormal (HP: 160-440, LP: 31-110 for 2-10 year olds; HP: 275 ± 122, LP: 51 ± 24 for 8-11 year olds, n = 14; (8)). The significantly elevated LP/HP ratio further confirms the diagnosis.

Patient 3.I (female):

Clinical Presentation and Medical History

Patient 3.I was last evaluated at age 5 years. She is the first child of non-consanguineous parents. Prenatal history was unremarkable until 38+5 weeks of gestation, when a primary cesarean section was performed due to breech presentation. She required brief oxygen supplementation postnatally but adapted well. Marked hypotonia and delayed motor development prompted early evaluation. Initial muscle ultrasound revealed increased echogenicity (Figure 1), raising suspicion of congenital myopathy. Over time, muscle weakness improved and clinical focus shifted to joint hypermobility, progressive kyphosis, and hypertrophic scarring.

Motor and Skeletal Findings

The patient achieved independent walking at 28 months. At age 5, she remains clinically stable. She uses orthopedic supports, including an SPIO vest and shoe inserts, and can

walk and run independently, though with reduced speed and coordination compared to peers. She has a mildly protuberant lower abdomen and shows hypertrophic scarring.

Genetic Diagnosis

Extensive genetic investigations—including karyotyping, arrayCGH, NGS panels for congenital myopathies, and targeted analyses for spinal muscular atrophy type 1 (SMN1 sequencing/CNV), Prader-Willi syndrome, and congenital myotonic dystrophy type 1—were negative. Trio exome sequencing at age 1.5 years initially failed to identify a diagnosis. Following the identification of *PLOD1*-related kyphoscoliotic Ehlers-Danlos syndrome (kEDS) in another individual, reanalysis of her exome data revealed a homozygous duplication of exons 10–16 in *PLOD1* (NM_000302.4), previously excluded due to stringent frequency filters.

Muscle Ultrasound

Longitudinal muscle ultrasound since infancy demonstrated persistently increased echogenicity in quadriceps femoris, soleus, adductor longus, gracilis, biceps brachii, and paravertebral muscles, with strand-like or nodular patterns suggestive of connective tissue proliferation. Over time, background echogenicity decreased, though fibrotic patterns persisted without progression. Normal muscle architecture became more apparent as muscle mass increased (Figure 1).

Vascular Imaging, Complications, and Outcomes

Vascular ultrasound of the heart, great vessels, and peripheral arteries age 5 years showed no aneurysmal changes. No clinical vascular events have been reported to date.

Urine pyridinoline analysis (in nmol/mmol creatinine) (HPLC measurement):

(HP) Pyridinoline/Creatinine: 93; (LP) Deoxypyridinoline/Creatinine: 478.3; LP/HP ratio: 5.14. The age-related reference values for collagen cross-linking were abnormal (HP: 160–440, LP: 31–110 for ages 2–10 years; HP: 343 ± 207, LP: 72 ± 50 for ages 5–8 years, n = 12; (8)). The significantly elevated LP/HP ratio further supports the biochemical profile characteristic of PLOD1-associated kEDS.

Patient 4.I (female):

Clinical Presentation and History

Patient 5.I is a 3-year, 5-month-old girl, last examined at this age. She is the first child of non-consanguineous parents. She was born at 36+2 weeks' gestation via cesarean section due to breech presentation, with a history of placental edge detachment and postnatal hypoglycemia. The patient was first evaluated at 5 months due to severe muscular hypotonia and delayed motor development. Mental and cognitive development is normal. Initially a congenital myopathy was suspected. Muscle sonography showed mild to moderate echogenic alterations in multiple muscle groups. By 8 months of age, persistent muscular weakness, kyphosis, and pronounced joint hyperlaxity were observed, raising suspicion for Ehlers-Danlos Syndrome (EDS). Trio-

exome sequencing did not yield a conclusive genetic diagnosis, leading to her inclusion in a genome-wide sequencing trial. At 9 months, a homozygous duplication of exons 10-16 in the PLOD1 gene was identified, confirming the diagnosis of kEDS.

Motor and Skeletal Findings:

The patient exhibits significant motor delays but has shown gradual progress. She achieved independent walking at 27 months, though her gait endurance remains markedly reduced, with fatigue occurring after walking 20-30 meters outdoors. On smooth indoor surfaces, she can walk at a slightly increased speed. Stair climbing remains impossible, and she frequently resorts to crawling for mobility. Bipedal and monopedal hopping is possible, though right-sided coordination appears slightly weaker. She rises from the floor through a prone position, suggesting mild axial weakness, but does not display a clear Gowers' sign. Musculoskeletal examination revealed generalized joint hypermobility with pronounced ligamentous laxity. The ankles exhibit extreme dorsiflexion. Hyperextensibility of the fingers and wrists is present, with dorsiflexion allowing near-contact of the fingers to the forearm. She has a flatfoot deformity with absent arch formation when toe-standing. No significant scoliosis or kyphosis was observed at the last examination. The skin is notably dry, soft, and hyperelastic, with hypertrophic scar formation.

Genetic Diagnosis:

Testing for regarding Spinal Muscular Atrophy Type 1 and trio genome sequencing revealed negative results. Following the diagnosis of another individual with PLOD1-kEDS, a query regarding homozygous carriers of intragenic *PLOD1* variants in the in-house genome database was performed. This led to reanalysis of the genome data with diagnosis of PLOD1-kEDS due to a homozygous duplication of exons 10–16 in *PLOD1*. The parents were heterozygous carriers of the variant.

Muscle Ultrasound:

Muscle ultrasound revealed varying degrees of echogenicity alterations, primarily affecting the medial hamstrings and biceps femoris, both of which exhibited moderate structural changes with increased echogenicity and reduced definition of intramuscular septa. Mild alterations were observed in the gastrocnemius, gracilis, and paravertebral muscles, as well as in the quadriceps femoris, which displayed a mixed pattern of normal and mildly increased echogenicity. Tibialis anterior, adductor longus, biceps brachii, triceps brachii, deltoid, and rectus abdominis, appeared normal.

Vascular Imaging, Complications and Outcomes:

Vascular ultrasound, last performed at the age of 2 years and 6 months, showed normal peripheral arteries and veins without aneurysmal changes. No vascular insults have been recorded to date. No significant cardiac abnormalities were noted in echocardiography.

Urinary pyridoline analysis:

Urinary pyridinoline cross-linking analysis confirmed an abnormally high LP/HP ratio, further supporting the diagnosis: HP Pyridinoline/Creatinine: 80; LP Deoxypyridinoline/Creatinine: 473.8; LP/HP ratio: 5.92. Age-related reference values for collagen cross-linking were abnormal (HP: 160–440, LP: 31–110 for ages 2–10 years; HP: 343 ± 207, LP: 72 ± 50 for ages 5–8 years (8)).

Patient 5.I: (female)

<u>Clinical presentation and medical history:</u>

Patient 5.I is the seventh child of consanguineous (first-degree cousins) parents of Syrian origin. One of her siblings died in the neonatal period and another pregnancy ended in late fetal loss. She was initially evaluated for pronounced muscle hypotonia and joint hypermobility at the age of 3 years, with differential diagnoses including kEDS or Ullrich type congenital muscular dystrophy/congenital myopathy. In addition, she had mild follicular hyperkeratosis and slightly bluish cornea. Other findings included thoracic kyphosis, a history of congenital calcaneovalgus feet with serial casting, and a submucous velar cleft leading to velopharyngeal insufficiency. In addition, she has a history of thalamostriatal vasculopathy identified on neonatal cranial ultrasound and a horseshoe kidney. Her cognitive and language development appears normal within the limits of a language barrier. She achieved independent walking at the age of 2.5-3 years. Chronic constipation was also noted.

She has a known history of epilepsy, with her first generalized tonic-clonic seizures occurring in January 2019 at the age of one year in the context of an infection. Additional seizures followed in May 2019 a few months later, and EEG recordings showed bitemporal, predominantly right-sided seizure activity. Anticonvulsant medication with levetiracetam was started and no further seizures occurred.

Initial external EEG recordings showed bitemporal seizure activity, predominantly on the right side. However, a control EEG in December 2021 was within normal limits without epileptiform activity. The patient had intermittent inspiratory stridor, which seemed to be exacerbated during infections and episodes of agitation. An ophthalmologic examination performed three months earlier at the age of five years was normal.

<u>Motor and skeletal findings:</u>

At six years of age, the patient´s walking distance is severely limited; she can walk 300-400 meters before experiencing pain or needing to rest. She has been fitted with new ankle-foot orthoses, which she wears with orthopedic shoes. The patient presents with a forward flexed stance and incomplete knee extension when walking. There is a pronounced kyphosis in the sitting position, although it is partially correctable. A left convex thoracic scoliosis is present and she is treated with a spinal brace. Joint hypermobility is noted, especially in the knees, elbows, thumbs, and small fingers, with a Beighton score of 8/9. Her teeth are irregularly aligned. Her ankles are valgus with standing and walking.

Genetic Diagnosis

Molecular genetic testing, including Trio WES, initially yielded unremarkable results. Re-evaluation as part of a re-analysis of the entire human genetics database at TUM after the diagnosis of patients 2.I, 3.I, and 4.I confirmed the diagnosis of kEDS by identifying a homozygous duplication affecting exons 10-16 of the PLOD1 gene.

Vascular Imaging, Complications and Outcomes:

Vascular ultrasound of other vessels was not performed. Cardiovascular evaluation remained unsuspicious so far.

Muscle ultrasound

At 3 years of age, there were no abnormalities in the thigh muscles. Increased fatty infiltration was suspicious in the muscles of the lower legs.

Urinary Pyridinoline Analysis:

Urinary pyridinoline cross-linking analysis confirmed an abnormally high LP/HP ratio, further supporting the diagnosis: HP Pyridinoline/Creatinine: 80; LP Deoxypyridinoline/Creatinine: 473.8; LP/HP ratio: 5.92. Age-related reference values for collagen cross-linking were abnormal (HP: 160–440, LP: 31–110 for ages 2–10 years; HP: 343 ± 207, LP: 72 ± 50 for ages 5–8 years (8)).

Patient 5.II (female):

Clinical Presentation and History

The female patient, last examined at the age of 5 years, is the fourth child of consanguineous parents (first-degree cousins) of Syrian origin. She is the first-degree cousin of patient 5.I. The pregnancy was reported to be uneventful, though the mother noted an absence of fetal movements. At birth, she presented with bilateral periorbital hematomas. A cranial ultrasound performed in Syria suggested the presence of an intracranial hemorrhage. The patient was initially evaluated due to pronounced muscular hypotonia and joint hypermobility. Last examination was age 3 years. She presented with thoracic kyphosis and developmental delay, particularly in motor milestones. She achieved independent walking at the age of 2.5 years. Her cognitive development appears appropriate for her age.

Genetic Diagnosis

A molecular genetic investigation confirmed the diagnosis of kEDS through the identification of a homozygous duplication affecting exons 10–16 of the PLOD1 gene. The diagnosis was suspected due to the positive family history and was therefore established promptly, as the condition had already been diagnosed in the patient's cousin at that time.

Motor and Skeletal Findings

The patient exhibits significant joint hypermobility affecting both large and small joints. Her gait remains unstable, and she experiences difficulty with prolonged standing and walking. At her last examination, a thoracic kyphoscoliosis was evident, and her joint hypermobility was pronounced, affecting her knees, elbows, fingers, and ankles.

Muscle ultrasound: not performed.

Vascular Imaging, Complications and Outcomes:

Bilateral orbital hematomas and suspicion of intracranial hemorrhage at birth. A vascular sonography was not performed. No significant cardiac abnormalities were noted in echocardiography.

Urinary pyridinoline analysis: not performed.

Patient 6.1 (female):

Clinical Presentation and History

This firstborn child of non-consanguineous parents, last examined at the age of 11 years, was referred at the age of 5 years to the neurologist due to suspected muscle weakness/congenital myopathy. The ophthalmological exam was normal by the age of 8 years. The patient is diagnosed with coeliac disease and asthma.

Motor and Skeletal Findings

She had a history of delayed motor development with independent walking age 2,2 years, muscular hypotonia and joint hypermobility, with recurrent subluxation of the shoulder. The patient was born with prominent spinous processes but no kyphosis was reported, however she suffered from scapula alata, pectus excavatum, a shortened Dig 5 of the hand and Dig 4 of the feet. At the age of 8 years, she was diagnosed with thoracolumbar scoliosis (16°).

Genetic Diagnosis

A molecular genetic investigation confirmed the diagnosis of kEDS through the identification of a homozygous duplication affecting exons 10–16 of the *PLOD1* gene. The diagnosis was suspected due to clinical findings and the significantly increased LP/HP ratio.

Molecular analysis: Amplification of PLOD1 by genomic PCR of the region between intron 9 and intron 16 and confirmation of the result on a second independently amplified PCR amplicon according to the protocol published in: Giunta et al., Mol Genet Metab 86:269-276, 2005.

Muscle Ultrasound: Not performed

Vascular Imaging, Complications and Outcomes:

At the age of 10 years, she suffered from sudden and severe lumbar pain, followed by persistent and heavy abdominal pain. The first line investigation by ultrasound revealed a dissection of the superior mesenteric artery, confirmed by CT and MRI within less than 2 hours. Under conservative management and immediate start with Celiprolol no ruptures occurred and no additional aneurysm was noted.
There was no vascular ultrasound executed. Echocardiographic examination revealed no structural or functional abnormalities.

Urinary Pyridinoline Analysis (in nmol/mmol creatinine) (HPLC measurement) (HP)

Pyridinoline/creatinine; 148; (LP) deoxypyridinoline/creatinine: 763; LP/HP ratio 5.16. (HP: 160-440, LP: 31-110 for 2-10 year olds; HP: 275 ± 122, LP: 51 ± 24 for 8-11 year olds, n = 14; (8)). The LP/HP ratio 5.16 is significantly increased compared to this of controls and similar to this of patients with PLOD1-kEDS.

Patient 7.1 (male)

Clinical Presentation and History: This child of consanguineous parents, last examined at the age of 17 years, was referred at the age of 4 months to the neurologist due to suspected muscle weakness. He was born at 40 weeks' gestation and exhibited pronounced neonatal hypotonia, joint hypermobility, distal arthrogryposis and cryptorchidism. He was diagnosed with a postpartum intraventricular hemorrhage (grade II) on the right side.

Motor and Skeletal Findings: The boy experienced a delayed motor development with independent walking at the age of 4 years. At the age of 17 years, he is able to walk about 10 minutes without rest. He is able to climb stairs with difficulties. Due to a severe scoliosis, spondylodesis was performed at the age of 12 years.

Muscle Biopsy: A muscle biopsy at the age of 1 ½ years revealed a pronounced myopathic pattern with significant caliber fluctuations and a slight increase in interstitial connective tissue.

Genetic Diagnosis: Array CGH inconclusive (age 1 year). Genome sequencing (age 15 years) revealed a large (4.8 kb) homozygous deletion on chromosome 1, including exon 1 of *PLOD1*.

Muscle Ultrasound: Not performed

Vascular Imaging, Complications and Outcomes: There was no vascular ultrasound executed. Echocardiographic examination revealed no structural or functional abnormalities.

Urinary Pyridinoline Analysis:
Pyridinolin: 107 nmol/mmol; Deoxypyridinolin: 868 nmol/mmol
